# Top‐Down Proteomics: Why and When?

**DOI:** 10.1002/pmic.202400338

**Published:** 2025-04-27

**Authors:** Philipp T. Kaulich, Andreas Tholey

**Affiliations:** ^1^ Systematic Proteome Research & Bioanalytics Institute for Experimental Medicine Christian‐Albrechts‐Universität zu Kiel Kiel Germany

**Keywords:** CE‐MS, LC‐MS, posttranslational modification, proteoform, proteoformics, quantification, splicing

## Abstract

Manifold biological processes at all levels of transcription and translation can lead to the formation of a high number of different protein species (i.e., proteoforms), which outnumber the sequences encoded in the genome by far. Due to the large number of protein molecules formed in this way, which span an enormous range of different physicochemical properties, proteoforms are the functional drivers of all biological processes, creating the need for powerful analytical approaches to decipher this language of life. While bottom‐up proteomics has become the most widely used approach, providing features such as high sensitivity, depth of analysis, and throughput, it has its limitations when it comes to identifying, quantifying, and characterizing proteoforms. In particular, the major bottleneck is to assign peptide‐level information to the original proteoforms. In contrast, top‐down proteomics (TDP) targets the direct analysis of intact proteoforms. Despite being characterized by a number of technological challenges, the TDP community has established numerous protocols that allow easy implementation in any proteomics laboratory. In this viewpoint, we compare both approaches, argue that it is worth embedding TDP experiments, and show fields of research in which TDP can be successfully implemented to perform integrative multi‐level proteoformics.

## 1 Introduction

The term proteome describes the ensemble of all proteins present at a given point in time and under defined conditions in a cell or organism. These temporal and condition‐dependent constraints highlight that a proteome is not static but highly dynamic, both in terms of the protein abundance as well as in the molecular form in which the proteins are present.

The protein sequence is encoded in the DNA. The genes are transcribed into RNA molecules, which are then translated into proteins. From a single gene, numerous different protein species [1] can arise due to various processes at the transcriptional and translational level, such as alternative splicing, alternative translation initiation sites, or co‐ and posttranslational modifications (PTMs). The various molecular forms in which a protein can exist are referred to as proteoforms [2], and the entirety of proteoforms originates from a single gene as a proteoform family [3]. Due to the combinatorial explosion caused by the manifold mechanisms contributing to their formation, the number of possible proteoforms outnumbers the number of encoding genes by far. For example, for human beings having a genome size of approximately 22,500 genes (a number which certainly still neglects numerous alternative and/or short open reading frames, which themselves also can form a plethora of proteoforms) [4], current estimates range from several million up to more than a billion possible different proteoforms [5, 6]; for single human cell lines, 250,000 unique proteoforms are estimated [7]. This creates a gigantic functional repertoire for any organism, for which methods of genome and transcriptome analysis are widely blind.

Therefore, the availability of information on the exact proteoform composition of a given proteome, including its dynamics in temporal and spatial dimensions, is critical to elucidate biological processes in detail since different proteoforms of a given protein can have different physicochemical properties and diverse molecular functions.

Protein modifications can result in changes in the protein structure or localization and, thus, interaction partners, finally resulting in different biological functions [8]. Up to now, a plethora of examples have been described highlighting the importance of actual proteoform information in various areas of life science, including a deeper understanding of human diseases, biomarker discovery, the assessment of biologics’ side effects, the development of targeted drugs, and diagnostics [9, 10, 11, 12]. For example, in Alzheimer's disease, various proteoforms (amyloid https://www.compart.com/en/unicode/U+1D6FD [Aβ] peptides) originate from the amyloid precursor protein by endogenous proteolytic cleavage events, such as two proteoforms that span residues 1–42 and 1–40, respectively [11]. However, only the Aβ 42‐proteoform is strongly correlated with Alzheimer's disease, but not the Aβ 40‐proteoform. Moreover, multiple sequence variants in the amyloid precursor protein result in different forms of Aβ, which can be either pathogenic or benign [11]. Therefore, to diagnose the disease, it is not enough to assess the overall abundance of Aβ at the protein level; it is essential to evaluate the abundance of specific proteoforms. Understanding the exact molecular composition of these proteoforms could also serve as a foundation for personalized medicine tailored to the various manifestations of Alzheimer's disease.

Mass spectrometry (MS)‐based proteomics is a key analytical approach for protein identification and quantification, enabling, for example, the investigation of proteome changes in response to various stimuli or the discovery of clinical biomarkers [13]. Moreover, specialized proteomics approaches allow, for example, the characterization of protein–protein and protein–drug interactions, the identification of PTMs, and the determination of protein localizations within tissues.

**FIGURE 1:**
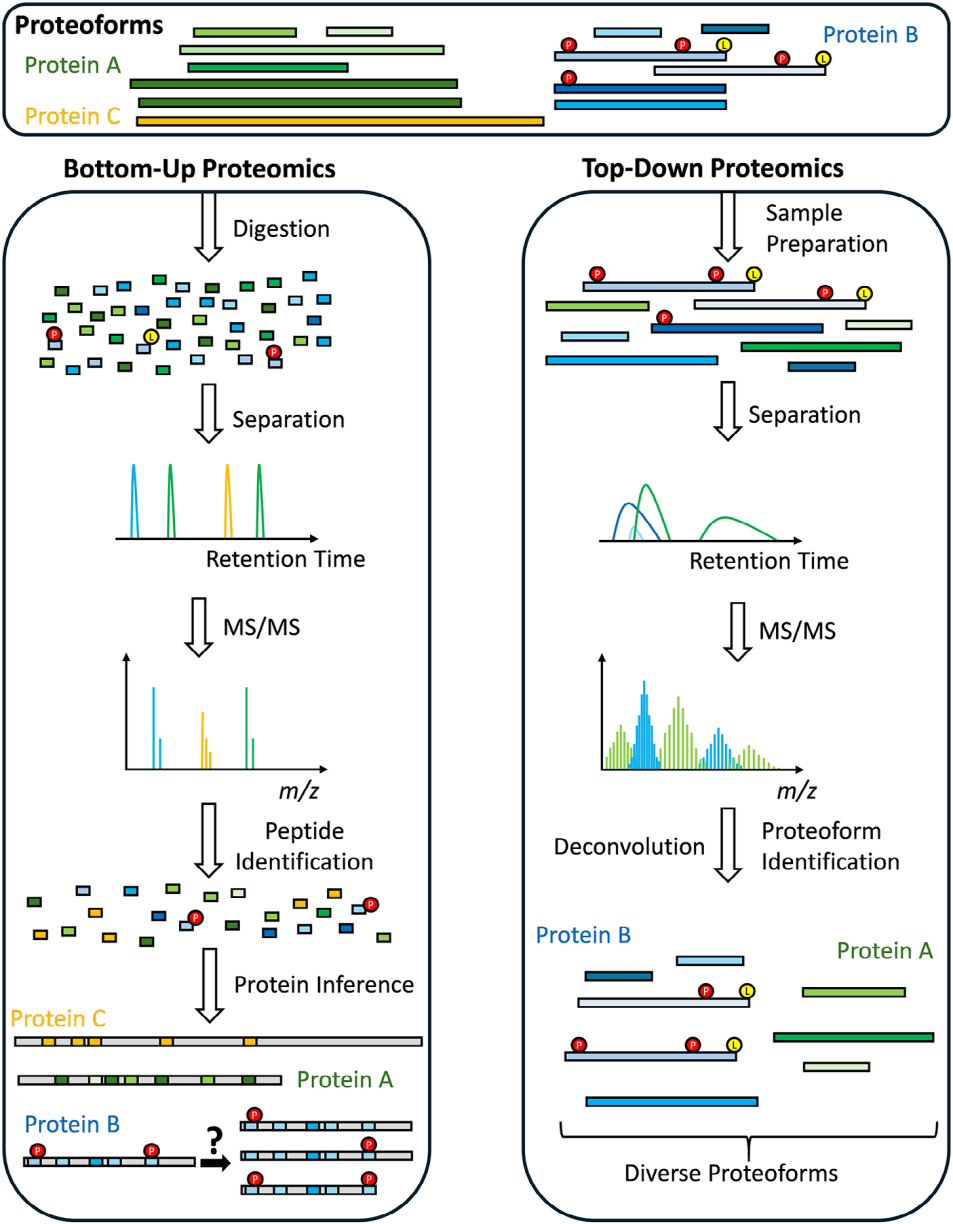
Schematic overview of BUP and TDP. The proteome consists of various proteoforms, which include all protein species derived from genes, encompassing transcriptional modifications, alternative translation sites, and co‐ or posttranslational modifications. The groups of protein species are colored green, blue, and yellow, respectively, and each represents proteoforms belonging to one proteoform family. In BUP (left), the proteoforms are digested into peptides, which possess relatively homogeneous physicochemical properties regarding size, charge, and hydrophobicity. This homogeneity allows the peptides to be easily separated and analyzed using MS. The small size of the peptides generates intense signals, and the monoisotopic peak can be easily identified. The peptides are identified by database search engines, and finally, protein groups are inferred. However, this process results in a loss of information, for example, due to the limited sequence coverage, and details about the original proteoforms (such as truncations or the PTM crosstalk) cannot be fully recovered. In TDP (right), intact proteoforms are analyzed. Typically, large proteoforms are depleted during the sample preparation. The separation of proteoforms prior to MS analysis can lead to broad elution profiles, resulting in complex MS spectra due to co‐eluting proteoforms. Furthermore, the high number of charge states and isotopologues results in crowded spectra and reduced sensitivity, particularly for large proteoforms. However, the advantage of TDP is that actual proteoforms, including all PTMs, are identified. BUP, bottom‐up proteomics; MS, mass spectrometry; PTM, posttranslational modification; TDP, top‐down proteomics.

In MS‐based proteomics, there are two main approaches for proteome analysis: Top‐down proteomics (TDP) and bottom‐up proteomics (BUP) [14]. While TDP targets the direct analysis of intact proteoforms, BUP involves enzymatic digestion of proteoforms into peptides, which are then analyzed, identified, and finally inferred into protein groups (Figure 1). Up to now, the vast majority of proteomics analyses have been performed using BUP, mainly due to the more homogeneous physicochemical properties of small peptides compared to large proteoforms, which facilitates the analysis in terms of separation, mass spectrometric detection, and bioinformatic identification. A compromise between BUP and TDP is middle‐down proteomics (MDPs), where larger peptides are generated, for example, by specialized proteases or chemical cleavage [15].

## 2 Bottom‐Up Proteomics Is Powerful ‐ But How About Proteoforms?

Liquid chromatography (LC)‐MS‐based BUP has become the gold standard in proteomics, and numberless, very powerful, sensitive, and fast approaches have been developed since the field was born at the beginning of the 2000s. Protein “identification” studies to decipher the dynamics of protein abundances and to analyze PTMs at a large scale are now standard worldwide and have strongly contributed to advancements in numerous fields, such as precision medicine and drug development. But how about the real functional currency [16] in cells—the proteoforms?

TDP targets the analysis of intact protein molecules, providing complete proteoform information and, thus, can theoretically elucidate the entire complexity of the proteome. In contrast, BUP inherently loses proteoform information due to the digestion of the proteoforms into peptides. This digestion creates the need for protein inference from peptide‐level data [17], a process that is well elaborated but also characterized by a number of fundamental drawbacks.

For example, the digestion of protein species/proteoforms with a high sequence similarity results in the generation of a high number of shared peptides, making it impossible to elucidate which peptide belongs to which proteoform. This problem is getting worse as, despite BUP being a highly sensitive approach, only a limited number of peptides are usually identified per protein, which per se leads to incomplete sequence coverage (Figure 1). This can be caused, for example, by the generation of very short peptides (which can not be unambiguously assigned to a protein) or peptide loss during sample preparation [18]. Moreover, modified peptides are often overlooked since they are typically not included in the database search to avoid dramatically expanding the search space. Even when data‐independent acquisition (DIA) approaches increase the number of peptide identifications, a large gap in obtaining complete sequence (including PTM) information still remains [18].

A further issue is assigning peptides with PTMs to a specific proteoform, especially when the latter is modified multiple times. Even if each identified peptide provides partial proteoform information, for example, information about a certain PTM, it is uncertain how multiple peptides of a given protein are linked to each other. A prominent example of this problem is the analysis of PTM crosstalk (that is, the combinatorial action of multiple PTMs) [19]. Suppose two modified peptides of a given protein are identified, then it cannot be distinguished from which proteoforms they were generated. Thus, it is ambiguous if there is only one proteoform of the protein in the sample carrying both modifications, two proteoforms with one or the other modification, or a mixture of these proteoforms (Figure 1). However, elucidating the PTM crosstalk is critical since it can influence a wide range of molecular factors, such as the proteoform structure, interaction partners, and biological function. Note that, compared to BUP, MDP has the advantage that the longer size of the peptides increases the likelihood that multiple PTMs may be identified, allowing for partial retaining proteoform information [19, 20]. However, like BUP, MDP faces the fundamental problem of protein inference, leading inherently to a loss of proteoform information.

All these factors finally lead to the fact that in BUP, protein groups are inferred, but the complete proteoform information is not accessible. This not only has consequences on the identification but also can lead to false conclusions regarding the quantification. For instance, an (inferred) protein may show no significant fold‐change in a quantitative BUP study as the peptide‐based information misses the presence of different proteoforms of the given protein, of which some are higher, but others are lower, differentially abundant [21, 22, 23]. For example, this situation was observed in two recent studies with the model organism *Caenorhabditis elegans*. Here, the 40S ribosomal protein S28 was inferred in a BUP experiment, showing no difference in abundance between control and challenge conditions (heat stress). However, in a follow‐up TDP study, 18 different proteoforms of this protein could be identified, of which one showed higher abundance and four proteoforms showed lower abundance under the challenge condition [24].

Despite these BUP‐inherent problems, in recent years, several techniques have been presented to obtain a higher degree of proteoform information from peptide‐level data. In semi‐top‐down approaches, the intact proteoforms are separated in the first dimension prior to digestion, and the information regarding the first‐dimensional separation is utilized to derive proteoform information [25]. For example, a first‐dimensional size‐based separation can provide information about truncated proteoforms [25, 26, 27]. Other bioinformatic approaches exploit peptide abundances in combination with correlation analysis to derive proteoform information from quantitative BUP data [21, 28]. However, it is not possible to retrieve the complete proteoform information after the proteoform has been digested. Moreover, these approaches typically require a high number of peptide identifications and, thus, extensive sample preparation (i.e., due to multiple fractionation schemes or proteases utilized) [29] and measurement time.

The knowledge about proteoforms is essential for a detailed exploration of molecular processes. Therefore, TDP is the preferred approach for gaining comprehensive insights into the proteome of a sample, as it inherently preserves the complete proteoform information.

## 3 When TD Promises to Be Superior in Proteoform Analytics, Why Is It Not Applied More? Present Limitations and To‐Dos Ahead!

Both BUP and TDP share, in principle, a very similar workflow, starting from sample preparation, followed by separation of the analytes by means of chromatographic or electrophoretic approaches, MS‐based detection and sequencing, and finally, data interpretation by means of bioinformatics approaches. In BUP, an essential additional step is the digestion of the proteins into peptides, which is performed as part of the sample preparation. So, where is the difference? Due to the characteristics of intact proteoforms, especially their highly elevated complexity and heterogeneity regarding their physicochemical properties compared to shorter peptides, it is clearly more than just omitting the digestion step (Figure 1). An excellent introduction and tutorial on the general issues to be taken into account when analyzing intact proteoforms was presented recently [30].

The analytical challenges increase with the increasing molecular mass of the analytes, and thus, the procedures at all levels of analysis have to be adapted to change from BUP to TDP [31]. A recent study described the influence of sample preparation approaches on the outcome of TDP experiments [32], also highlighting potential pitfalls overseen in BUP analysis due to the inherent loss of proteoform information described above.

Efficient proteoform fractionation and separation are critical to decrease co‐elution and ion suppression effects, resulting in an improved depth of TDP analyses [33]. Despite significant advancements made in recent years, the field of intact protein separation by LC [34] or CE [35, 36] remains a challenging task and will certainly profit from future developments in order to reach the power of contemporary peptide‐level separation.

The same holds true for the entire subject of MS. A significant limitation is the reduced sensitivity of (large) proteoforms in TDP compared to (small) peptides in BUP. The larger the analytes, the higher the number of isotopes, and the more charge states are typically observed in electrospray ionization. This isotopic and charge dilution effect inherently results in lower signal‐to‐noise ratios and, thus, reduced sensitivity, as the signal of a single analyte is distributed to multiple peaks [37]. Moreover, with increasing proteoform size, the complexity of both the MS1 (due to multiple isotopic peaks of multiple charge states) and MS2 (due to a higher number of theoretical fragment ions) spectra increases. This high spectral complexity leads to particular requirements in data acquisition (e.g., on‐the‐fly deconvolution [38]) and data interpretation (e.g., accurate determination of the monoisotopic mass [39, 40] and sensitive database search algorithm [41, 42]). To this end, typically, high‐resolution mass spectrometers are utilized to resolve the isotopes of large proteoforms, and multiple spectra are summed to increase the signal‐to‐noise ratio, resulting in lower acquisition speed. Moreover, crowded fragment spectra resulting from the high number of fragment ions lead to limited residue cleavage, complicating the exact localization of modifications.

Furthermore, an important field requiring special attention in the future development of TDP will be data validation, for example, the development of mechanisms that facilitate the recognition of false‐positive identifications. First approaches, for example, for the classification of quality of proteoform identifications, have been implemented [43]. Moreover, the Characterization Score (C‐score) has been developed, providing a quality measure for the characterization confidence of highly related proteoforms [44]. Here, the field of TDP will certainly see the same learning curve as was seen in the evolution of BUP.

As a result of these limitations and still to be filled gaps, compared to BUP, TDP at the present moment is characterized by (i) lower sensitivities, (ii) reduced throughput, (iii) an upper mass limit for (routine) in‐depth analyses, and (iv) limitations in the precise localization of modifications. Note that all challenges faced by TDP are more severe the larger the proteoforms and the more complex the sample [45]. Typically, in current TDP studies, hundreds to thousands of proteoforms are identified, predominantly under 30 kDa and derived from the most abundant proteins.

However, a number of concepts have been successfully introduced that hold the potential to improve the situation in the future [45]. For example, it is possible to extend the accessible mass range by using specialized techniques, such as proton transfer charge reduction [46] or by analyzing samples with lower resolution, such as in the medium/high acquisition approach [47]. Moreover, charge‐detection MS, which allows for the determination of the *m/z* ratio as well as the charge state from individual ions, increases the accessible mass range as well as the sensitivity [48, 49].

In order to simplify the complexity of mass spectra and increase the quality of identifications, gas‐phase fractionation strategies have proven to be highly effective. For instance, high‐field asymmetric waveform ion mobility spectrometry significantly increases the number of identifications achieved within the same measurement time [50, 51]. Moreover, the gas‐phase separation of fragment ions, potentially utilizing techniques such as trapped ion mobility spectrometry, could drastically enhance the sequence coverage [52]. Increased throughput can be accomplished through the use of faster mass analyzers, improved chromatography, and optimized data acquisition strategies (i.e., by efficiently fragmenting diverse proteoforms).

## 4 When and How You Should (and Easily Can) Integrate Top‐Down Proteomics in Your Projects: A Motivation Letter

The above‐mentioned (incomplete) list of limitations and analytical “still to be dos” in TDP shows that this approach still lags behind the power of BUP. On the other hand, TDP has left its early infancy and has now reached its adolescence—becoming more and more powerful but also sometimes difficult to handle. Moreover, there is no doubt that we need to position our telescopes towards the proteoforms rather than on inferred protein groups when we really want to understand the language of life, keeping the limitations of our present gold standard, BUP, in our minds. How do we cope with this situation?

The majority of the proteoforms identified (and quantified) in present TDP studies have masses below approximately 30 kDa [45]. In many cases, the studies performed a prefractionation step to enrich the low molecular weight fraction or deplete the high mass fraction, resulting in an a priori loss of larger proteoforms [32].

However, note that, in this subproteome mass range from 0–30 kDa, numerous biologically relevant proteins (calculated from their genome encoded sequences) are present, and approximately 28% and 50% of all reviewed human and *Escherichia coli* proteins, respectively, deposited in UniProt [53] are smaller than 30 kDa. Moreover, several pathobiologically relevant proteoforms are in this mass range, such as histones [54], proteoforms of the KRAS gene (the most frequently mutated oncogenes in human cancer) [55], or the Aβ peptide relevant in Alzheimer's disease [11].

A class of proteins that is very well accessible for TDP is microproteins and short open reading frame‐encoded peptides (SEPs), which are typically built by less than 100 amino acids. Recently, TDP has elucidated that SEPs can be present in diverse proteoforms [4]. Moreover, peptidomics, that is, the analysis of endogenous peptides in biological systems, is closely related to TDP since typically intact proteolytically processed proteoforms are identified [56, 57].

How to start a TDP experiment? With the availability of easy‐to‐implement TDP workflows (including sample preparation, chromatographic separation, mass spectrometric acquisition, data analysis, and evaluation) [32, 39, 41, 58–61], the barrier to starting with TDP for classical proteomics laboratories is relatively low. Numerous TDP sample preparation protocols have been presented in the last few years that do not require specialized equipment and can often be already performed in a standard proteomic laboratory. An example is the PEPPI protocol (passively eluting proteins from polyacrylamide gels as intact species) [62], which is based on proteoform separation by SDS‐PAGE and subsequent elution from the gel, enabling pre‐fractionation of targeted mass ranges and even provides the chance for quantitative analysis [63, 64]. For data analysis, there are a variety of easy‐to‐use and high‐quality open‐source software packages, such as FLASHDeconv [39] for deconvolution, FLASHQuant [65] for quantification, and TopPIC for proteoform identification [41].

Apparently, analytical challenges seem to demand high‐end instrumentation when it comes to detailed information; however, numerous examples impressively show that this is also possible with standard equipment available in almost every proteomics lab. Most mass spectrometers are capable of performing TDP experiments, including high‐resolution Orbitrap and Fourier‐transform ion cyclotron resonance mass spectrometers, as well as low‐resolution ion traps and time‐of‐flight mass spectrometers [66, 67, 68]. For LC separation, the choice of the stationary phase is an important point. Here, the C18 reversed phases usually applied in BUP should be replaced, for example, by C4 materials or similar columns with lowered hydrophobicity, which is also easy to accomplish and can serve as a good starting point for entering the field of TDP.

While the analytical challenges increase with proteoform mass, and the above‐mentioned current routinely accessible mass range lies presently below approximately 30 kDa (including already a variety of biologically highly relevant proteoforms), this should not prevent the search for ways to extend the application of TDP to the larger mass proteins. For example, the field called “native Proteomics” presently deals with much less complex samples but large proteins or even large complexes and utilizes comparable analytical approaches. Recently, online coupling of capillary zone electrophoresis with an Orbitrap mass spectrometer enabled the detection of 72 proteoforms or protein complexes in a mass range of 30–400 kDa from a complex *E. coli* lysate [69], highlighting the potential for analyzing large proteoforms. It can be expected that future advancements, such as improved liquid‐ or gas‐phase separations, more sensitive mass spectrometers, and enhanced algorithms for deconvolution and proteoform identification, will gradually extend the current mass limit of TDP towards higher proteoform masses.

## 5 Closing the Gaps: Integrative Multi‐Level Proteoformics

With the current tools at hand, we highly encourage the proteomics community to integrate TDP in their standard projects, that is, perform TDP in addition to classical BUP‐based experiments if enough sample material and resources are available. The actual identification of proteoforms using TDP inevitably leads to a gain in knowledge compared to BUP and can provide insights that would otherwise not be recognized.

Moreover, the integration of the data derived from different proteomics approaches, including BUP, TDP, MDP, and terminomics [70] approaches (“integrative multi‐level proteoformics”) holds the potential to create enormous additional knowledge about the functional potential of a given proteome. While this integration still requires improvements of existing and the development of novel bioinformatics tools, it will enable the study of important biological mechanisms, such as regulatory processes triggered by the crosstalk of PTM.

Further, with the broader application of TDP experiments in the proteomics community, this will certainly also be fruitful for driving this approach more and more forward, paving broad highways towards the understanding not only of the alphabet but of the words and sentences forming the molecular language of life: the proteoforms.

## Conflicts of Interest

The authors declare no conflict of interest.

## Data Availability

The authors have nothing to report.
